# Application of artificial intelligence in diagnosis and management of fetal growth disorders: a comprehensive review

**DOI:** 10.3389/fmed.2025.1737391

**Published:** 2026-01-16

**Authors:** Franciszek Ługowski, Julia Babińska, Paweł Jan Stanirowski

**Affiliations:** 1 1st Department of Obstetrics and Gynecology, Medical University of Warsaw, Warsaw, Poland; 2 Doctoral School, Medical University of Warsaw, Warsaw, Poland

**Keywords:** artificial intelligence, fetal growth disorders, fetal growth restriction, fetal macrosomia, intrauterine fetal growth restriction, large-for-gestational-age, small-for-gestational-age

## Abstract

Fetal growth disorders, including both fetal growth restriction and macrosomia, remain major contributors to perinatal morbidity and long-term health risks in adulthood. While ultrasound is the most frequently employed technique for the diagnosis of intrauterine growth abnormalities, its efficacy is constrained by the operator’s experience and variable accuracy. This review explores the role of artificial intelligence (AI) in advancing the detection and management of fetal growth disorders. We conducted a comprehensive literature search of major databases to identify original and review articles addressing the use of AI in fetal growth restriction, small-for-gestational-age and large-for-gestational-age fetuses, as well as fetal macrosomia. The available evidence indicates that AI models combining maternal, fetal, and imaging data exhibit a level of accuracy comparable to that of experienced clinicians, while also enhancing operational efficiency and reducing variability. Emerging applications include automated biometry, prediction models based on biomarkers and Doppler indices, as well as deep learning algorithms applied directly to ultrasound scans. These methods not only enhance diagnostic precision but also expand access to high-quality prenatal care, particularly in low-resource settings. Nonetheless, most of the published studies remain limited by retrospective designs, small sample sizes, and a lack of external validation. Addressing these challenges, along with ethical, technical, and regulatory considerations, will be essential for clinical translation. In conclusion, AI has the potential to become a cornerstone of precision perinatal medicine by enabling earlier diagnosis, individualized monitoring, and thus improved outcomes for both mothers and infants.

## Introduction

Biometric measurements conducted by means of ultrasound play a pivotal role in estimating fetal birth weight, hence enabling the assessment of the intrauterine fetal growth (1–3). Fetal growth restriction (FGR), small-for-gestational-age (SGA), and large-for-gestational-age (LGA) fetuses, as well as fetal macrosomia are examples of growth abnormalities routinely identified via ultrasonographic examination (4–6). Despite being a widely available, safe, and low-cost method, the diagnostic precision of ultrasonography is contingent upon the clinician’s proficiency, frequently resulting in considerable variability in measurements, which ultimately culminate in misdiagnoses and superfluous medical procedures (7). Moreover, since maternal characteristics such as obesity, diabetes, hypertension, nicotine use, and autoimmune diseases are recognized contributors to compromised fetal development *in utero*, their analysis is equally essential for establishing a successful prenatal diagnosis (8–11).

In 2020, global estimates indicated 23.4 million SGA births (12). Although the term refers purely to a fetal size definition, i.e., estimated fetal weight (EFW) below the 10th percentile, and includes constitutionally small but otherwise healthy infants, SGA fetuses are at an increased risk of neurodevelopmental impairments, metabolic syndrome, and pulmonary disorders in the adolescence and adulthood (13–15). In contrast, FGR implies a pathological limitation of growth, diagnosed not only by small size (often EFW < 3rd centile) but also by abnormal growth velocity or Doppler evidence of placental insufficiency and fetal compromise (16, 17). This distinction means that screening must go beyond biometric percentiles to include Doppler waveform analysis and longitudinal growth trajectory, as many SGA fetuses are not truly growth-restricted.

At the same time, the opposite end of the growth spectrum has become increasingly relevant: over the past few decades, the prevalence of LGA infants has risen to 15–20% in developed countries (18). Additionally, fetal macrosomia ≥4,000 g affects approximately 15–20% of fetuses in pregnancies with concomitant gestational diabetes mellitus and 40–45% with type 1 diabetes mellitus (19, 20). Beyond the complications at delivery, such as shoulder dystocia and birth trauma, infants born LGA or with macrosomia also carry a greater likelihood of developing long-term cardiometabolic disorders, including obesity, type 2 diabetes, and hypertension later in life (21, 22).

Considering all of the above-mentioned evidence, an early and accurate diagnosis based on the ultrasound measurements, maternal characteristics and other potentially useful factors (i.e., biochemical, metabolomic or genetic markers), is of utmost significance in the prenatal management of abnormal fetal growth. As a result, there is a need for cutting-edge tools that can aid physicians in this process.

**Figure 1:**
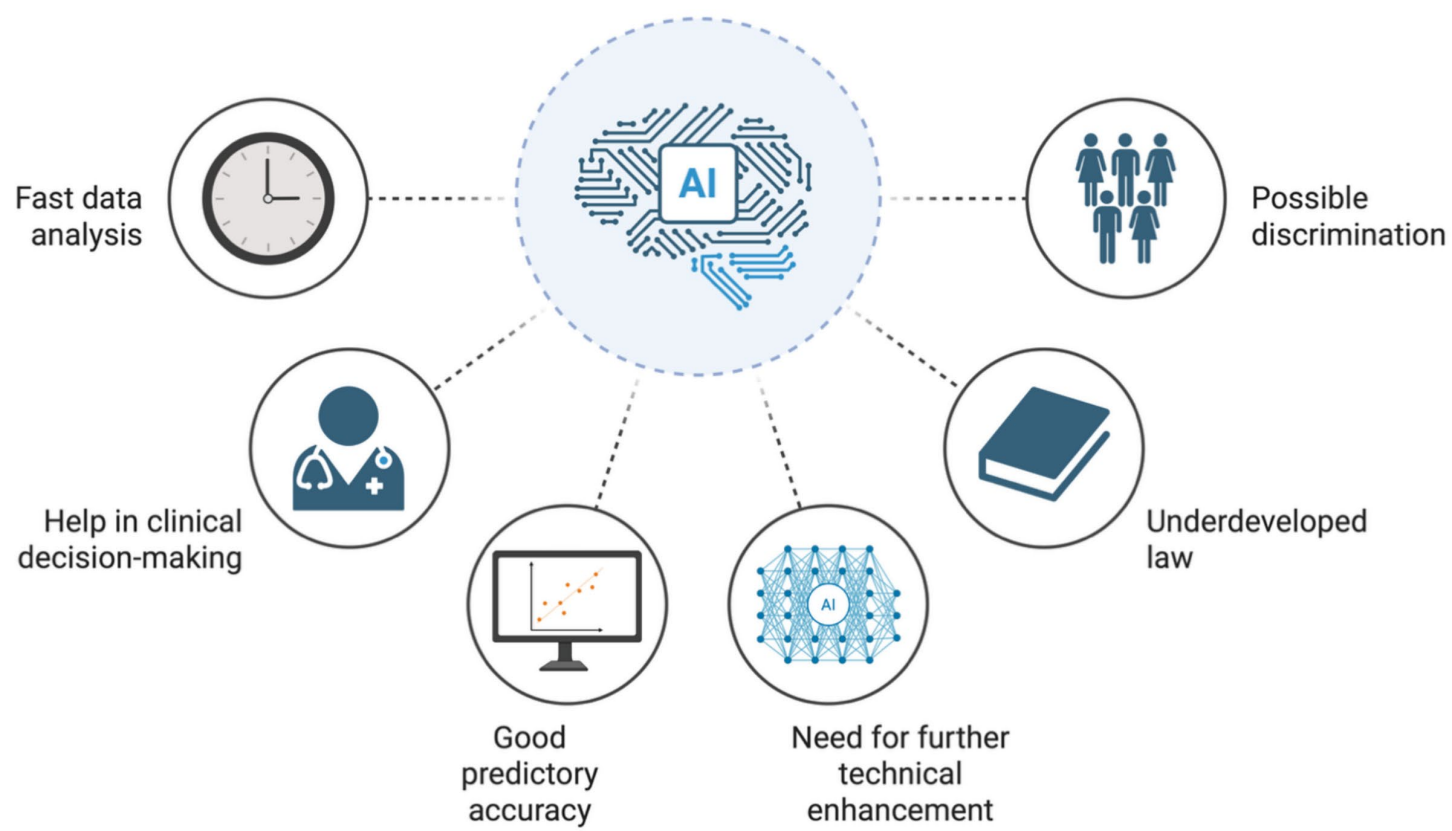
Advantages and limitations of AI application in fetal growth disorders. Created with biorender.com.

It has been suggested that the implementation of specifically developed artificial intelligence (AI) applications holds the promise for enhancing the prenatal detection of fetuses affected by intrauterine growth disorders. Such advancements have the potential to refine antenatal management strategies, thereby improving both perinatal care as well as short- and long-term neonatal outcomes. AI-driven technologies could transform the clinical approach to the aforementioned conditions by facilitating earlier and more accurate identification of high-risk pregnancies. Nowadays, this field is evolving dynamically in parallel with technological advancements, which translates into an increasingly profound knowledge base of AI in the domain of obstetrics and gynecology (23). Through the integration of advanced computational algorithms, AI systems are capable of discerning intricate patterns within maternal, fetal, and placental datasets that may elude conventional diagnostic modalities. This capability may enable more accurate risk stratification and the timely initiation of appropriate interventions. Additionally, by minimizing inter-operator variability and optimizing the allocation of healthcare resources, AI may play a pivotal role in the evolution of precision perinatal medicine (24). Finally, automated support in therapeutic decision-making and outcome prediction may facilitate the implementation of more time-efficient and safer strategies, which could be incorporated into future clinical guidelines. Nonetheless, currently adoption of AI remains limited owing to the high cost of implementation and the absence of sufficient technical infrastructure (Figure 1).

This review summarizes selected AI algorithms with potential utility in the diagnosis and management of fetal growth abnormalities, emphasizing their prospective impact on advancing personalized medicine. While prior reviews have largely focused on isolated aspects of AI in obstetrics, such as ultrasound biometry or the prediction of FGR, our review aims to provide a unique, integrative perspective across the entire spectrum of fetal growth disorders, including SGA, FGR, LGA, and fetal macrosomia. By synthesizing evidence from ultrasound imaging, maternal factors, Doppler studies, metabolomics, and genomic approaches, we highlight not only the current performance of AI models but also their translational challenges.

## Materials and methods

A comprehensive literature search was conducted between 1st March and 31st May 2025 without restrictions on the publication date. Relevant studies were identified through searches of the PubMed, Scopus, and Embase databases. The search strategy involved combining terms related to AI in fetal growth disorders and their available treatment modalities using the Boolean operator “OR.” Keywords included: *artificial intelligence, machine learning, deep learning, neural networks, predictive modeling, fetal growth disorders, small for gestational age, SGA, large for gestational age, LGA, fetal macrosomia, fetal growth restriction, FGR, intrauterine growth restriction, IUGR, prenatal diagnosis, obstetric ultrasound, perinatal outcomes*.

This article was designed as a narrative review; however, a structured approach was adopted to identify relevant publications. Inclusion criteria encompassed original studies investigating the application of AI in the diagnosis and management of fetal growth disorders, written in English, including prospective and retrospective analyses, clinical trials, reviews, and meta-analyses. Exclusion criteria comprised studies unrelated to the scope of this review, non-English publications, conference abstracts, and documents categorized as technical reports, editorials, letters, or duplicates.

### Evaluation of fetal biometry

Fetal biometry allows for the measurement of specific anatomical parameters to evaluate intrauterine fetal growth and development. The integration of AI into this process has the potential to substantially improve the precision, consistency, and efficiency of measurements, all of which are essential for antenatal surveillance.

Key biometric parameters utilized in the assessment of fetal growth include:

- Biparietal Diameter (BPD): the transverse diameter of the fetal head,
- Head Circumference (HC): the measurement of the perimeter of the fetal skull,
- Abdominal Circumference (AC): the circumference of the fetal abdomen, and
- Femur Length (FL): the linear measurement of the femoral diaphysis (25).

**Figure 2:**
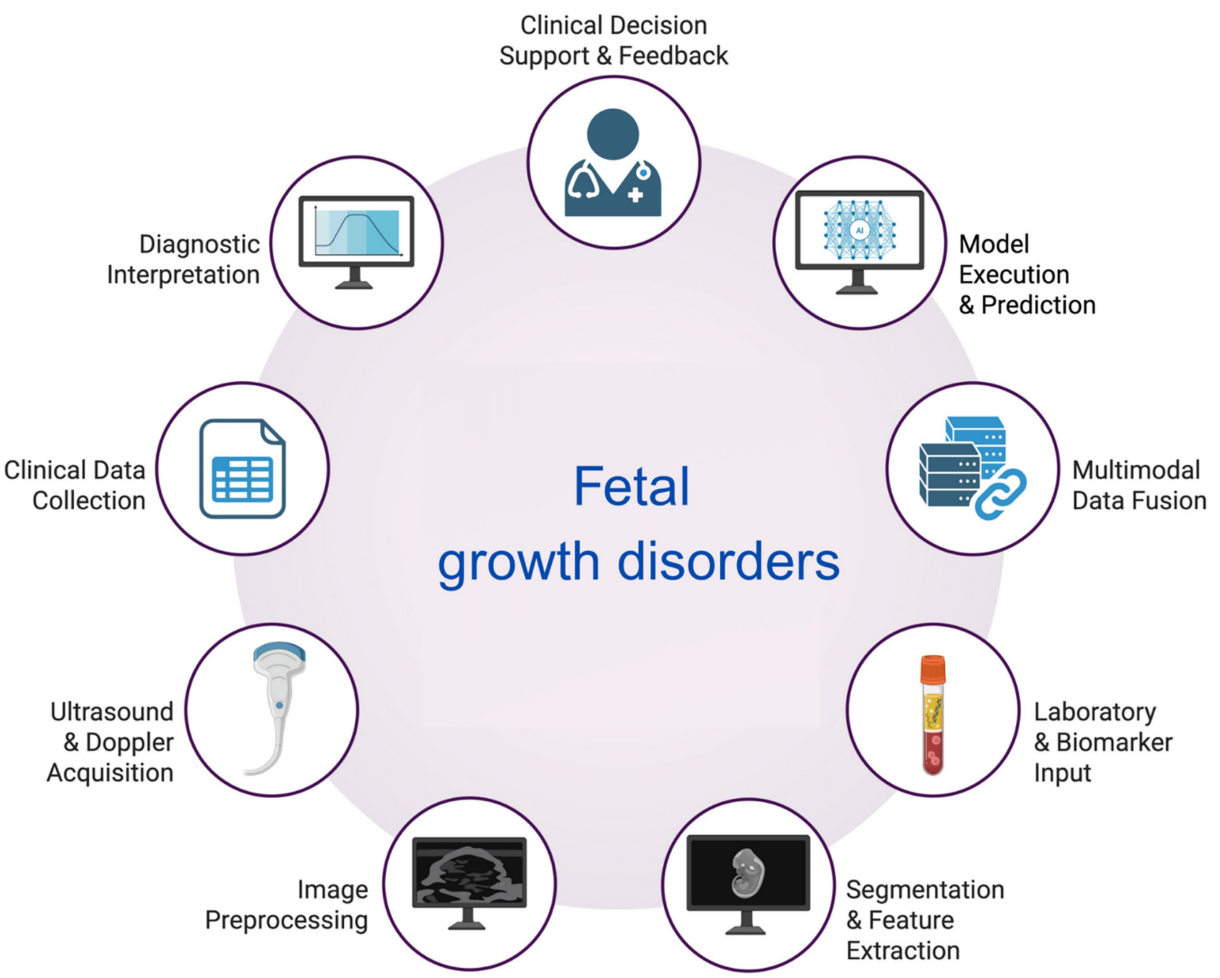
The workflow of AI usage in the assessment of fetal biometry and the diagnosis of fetal growth disorders. Created with biorender.com.

AI-based models, ranging from convolutional networks to random forests, have consistently achieved expert-level accuracy across multiple biometric parameters, including HC, AC, and FL (26, 27). Moreover, novel algorithms enable the automated segmentation of fetal anatomical structures in ultrasound imaging, thereby minimizing operator dependence and enhancing the consistency of biometric measurements (28). In concordance, deep learning (DL)-based segmentation tools can accurately delineate the contours of fetal organs and skeletal structures, thereby supporting precise and reliable biometric evaluations (29). The observation that in pregnancies complicated by maternal diabetes, AI-assisted risk modeling may allow for early detection of fetal overgrowth is another aspect of clinical relevance (30) These findings suggest that the implementation of AI can enhance clinical workflows by reducing interobserver variability, shortening examination times, and increasing diagnostic confidence. Together, these benefits position AI as a tool to improve both efficiency and reliability in fetal growth assessment; however, external validation remains limited (31–34). The AI-assisted workflow, including fetal biometry and other data useful in the prediction of impaired fetal growth, is shown in Figure 2.

Ghelich Ogli et al. (31) developed a convolutional neural network architecture designed to automatically measure BPD, HC, AC, and FL parameters from fetal ultrasound images with a Dice Similarity Coefficients (DICE) ranging from 0.84 to 0.98. The proposed model, utilizing relatively large datasets, along with an appropriate data augmentation algorithm, yielded satisfactory results with clinically acceptable errors (31). Venturini et al. (32) also demonstrated AI’s capability of accurate real-time biometric measurements based on fetal ultrasonography. In that study, AI-assisted scanning was performed with a mean time of 14.32 ± 2.85 min. Compared to manual assessment – 21.93 ± 3.24 min, *p* < 0.0001. Of importance, no statistically significant differences were noted between the manual and AI-assisted measurements for any of the standard parameters, except for the HC (31). Lastly, Płotka et al. (33) developed an AI model for fetal weight estimation that achieved a 3.75% deviation from human calculation, using solely an ultrasound video scan of the fetal abdomen. In that study, the mean absolute percentage error of AI-assisted birth weight estimations reached 2.59% ± 1.11% when compared with four senior clinicians using standard fetal measurements. Notably, observed intraclass correlation coefficients (ICCs) ranged from 0.9824 to 0.9872, indicating excellent agreement and performance comparable to expert human estimations (33). Supportive of the above-mentioned findings, AI’s clinically acceptable accuracy, combined with speed of measurement, was confirmed in several other studies (32, 34).

Numerous enhancements in the field of AI-assisted biometry have recently been introduced, proving AI’s exponentially growing application in obstetrics. For instance, BiometryNet, a tool designed to automate fetal biometry for multiple fetal structures using direct landmark detection, has resulted in reduced variability and improved landmark localization, thereby providing more accurate biometric measurements compared to commonly utilized methods (35). Furthermore, a novel AI model for measuring fetal intracranial structures during a 2D ultrasound was recently established in China (36). In that study, AI achieved very good consistency with manual measurements (average errors ranging from 0.29 to 3.57 mm and DICE averaging 90.32 and 92.18%), with an average measurement time of 0.49 s, which was more than 65 times faster compared to the traditional method (36). Finally, the application of AI has the potential to aid the obstetric populations in low-income countries. A pilot study has commenced in Uganda using the AI-based ScanNav FetalCheck software to enable accurate dating of pregnancies without the need for specialist sonographers (37). In order to assess the gestational age efficiently the software has been trained on a database of millions of ultrasound images evaluating fetal biometric measurements.

To conclude, current AI algorithms achieve accuracy comparable to experienced clinicians, with error margins within clinically acceptable ranges, while dramatically reducing the time required for assessment. These tools not only enhance diagnostic precision but also expand access to quality prenatal care in low-resource settings. It must be acknowledged, however, that even AI-augmented fetal biometry measurements based on standard ultrasound parameters have certain limitations. For example, in both pre-existing and gestational diabetes mellitus, maternal metabolic derangement and fetal hyperinsulinemia lead to soft tissue overgrowth, while standard fetal biometry primarily assesses osseous structures (38, 39). Consequently, tools or formulas used for the estimation of the fetal birth weight incorporating exclusively standard biometric parameters may be subject to measurement bias in diabetes-complicated pregnancies.

### Fetal growth restriction (FGR)

FGR refers to a fetus that fails to achieve its genetically predetermined growth potential, often due to underlying pathology, such as placental insufficiency, nutritional deficiencies, hypertensive disorders, or fetal abnormalities (11). According to the Delphi consensus, the diagnosis of FGR is made based on ultrasound EFW or AC measurement <10th percentile for gestational age, abnormal Doppler findings, or a significant decline in fetal growth velocity (11, 16).

The multifactorial pathogenesis of FGR implies that an ideal diagnostic tool should combine maternal characteristics, genetic/ biochemical data, in conjunction with fetal biometry and Doppler waveform evaluation. Contrary to traditional methods of assessment, machine learning (ML) algorithms are trained on large datasets of maternal and fetal parameters (24, 40). Deval et al. (41) analyzed maternal characteristics and biochemical markers utilizing Support Vector Machine (SVM) and Multilayer Perceptron (MLP) models in FGR detection. Their findings demonstrated a predictive accuracy of 95.5 and 88.5% with the SVM and the MLP algorithm, respectively. In addition, Bahado-Singh et al. (42) developed an algorithm for FGR prediction, where the correlation-based feature selection (CFS) method selected a metabolite panel that achieved an area under the receiver operating characteristic curve (AUC) of 0.90, with a sensitivity of 0.87 and specificity of 0.83. In that study, the AI-driven algorithm detected numerous altered metabolic pathways that positively correlated with the FGR, including beta oxidation, oxidation of branched-chain fatty acids, the urea cycle, phospholipid biosynthesis, lysine degradation as well as fatty acid, tryptophan, and methionine metabolism (42). Nonetheless, although the model achieved high accuracy, it was tested on a relatively small retrospective dataset and without external validation, which limits its generalizability (42). Other AI models leveraging biochemical markers, including networks trained on placental proteins, maternal metabolites, and inflammatory cytokines, have consistently shown high predictive accuracy for FGR, often exceeding 85–90% (41–45). Despite methodological differences, these approaches highlight the utility of combining maternal characteristics and molecular data with machine learning for more accurate FGR diagnosis.

Numerous models that employ clinical data to improve the prediction of FGR have been developed to date. In the study by Lee et al., the authors used easily accessible variables, including age, parity, underlying diseases, reproductive history, physical examination results, family history, laboratory results, and obstetric history, in the first and third trimesters of pregnancy, to establish a prediction model for FGR (46). The final cohort included 32,301 women, based on whom the predictive models achieved an AUC ranging from 0.73 to 0.78 in the training set and an AUC of 0.62–0.73 in the test set (46).

Besides maternal characteristics and fetal biometry, abnormal blood flow in the maternal-placental-fetal unit is of crucial importance for the FGR diagnosis and management. ML approaches applied to uterine or umbilical artery Doppler waveform indices, achieved strong discriminatory performance, with certain models exceeding 90% accuracy. Notably, the highest accuracy was achieved by the classification via regression (CVR) model (90.6%), which combines a pruned decision tree with smoothed linear models (47). A detailed analysis performed by the authors revealed that the model employing the lowest uterine artery pulsatility index (UtA-PI) was more accurate compared to the mean UtA-PI-based models (47). Another ML model that was recently developed by Gómez-Jemes et al. (48), utilized maternal and neonatal characteristics, Doppler measurements of the uterine artery indices, together with serum biomarker concentrations, such as soluble fms-like tyrosine kinase-1 (sFlt-1), placental growth factor (PLGF), and the sFlt-1/PlGF ratio. Based on these parameters, a modified decision tree classifier proved to be the most accurate, with a recall metric of 0.89 and an AUC metric of 0.87 (48). In accordance with the above-mentioned data, numerous other studies demonstrate that combining Doppler with maternal and neonatal variables improves AI predictive power, underscoring the importance of multimodal integration (47–50).

Regarding the application of genetic data for precise FGR diagnosis, two models analyzing nucleosome profiling data from cell-free DNA (cfDNA) have been developed (49, 51). Analysis of their results suggests that nucleosome profiling and promoter-based classifiers can predict FGR with moderate accuracy (AUC ~ 0.78–0.80). Xu et al. (51) used an SVM-LR model to utilize low-coverage whole-genome sequencing data from 810 pregnant women. A 14-gene combination achieved the highest accuracy after leave-one-out cross-validation (LOOCV) with an AUC of 0.8 and an accuracy of 81.8% in the SVM model, whereas a 12-gene combination reached an AUC of 0.776 and an accuracy of 81.1% in the LR model. Similarly, Guo et al. (49) presented a model using whole-genome sequencing of plasma cfDNA, which attained an accuracy of 78.9% in FGR prediction. In that study, promoter profiling-based classifiers were used to predict FGR in early gestational age utilizing the LR model (49). Collectively, these studies illustrate the feasibility of using genomic signals for fetal growth assessment, though their reliance on retrospective datasets and lack of prospective validation remain key limitations (49, 51).

Summing up, AI and ML have shown promising utility in improving the prediction of FGR by analyzing diverse data sources, including clinical parameters, ultrasound scans, biochemical markers, Doppler indices and genetic screening. Models using placental biomarkers, maternal serum metabolites, and gene expression data have achieved high accuracy (80–95.5%) in identifying pregnancies at risk of FGR. In addition, Doppler-based models incorporating UtA-PI and fetal hemodynamics have further enhanced predictive performance, with some algorithms reaching over 90% accuracy. Finally, large-scale clinical datasets have supported the development of trimester-specific models integrating maternal demographics, obstetric history, and laboratory results. These findings highlight the growing potential of AI in enhancing early detection and personalized management of FGR across various stages of pregnancy.

### Small-for-gestational-age (SGA) fetus

According to the definition, the diagnosis of SGA is determined solely by a statistical deviation of fetal size relative to a reference population. This is commonly considered to be present when EFW falls below the 10th percentile of standardized reference charts (52, 53). Notably, around 40% of fetuses with an estimated weight below the 10th percentile are constitutionally small but otherwise healthy (53). As a result, SGA represents variation in fetal size rather than a pathological condition per se. In contrast, FGR constitutes a pathological state frequently resulting from hypoxia or insufficient nutrient supply, without necessarily being associated with reduced fetal weight. For instance, a fetus classified as appropriate-for-gestational-age (AGA) may still be growth-restricted if its expected growth trajectory was intrinsically higher. As a consequence, equating FGR with SGA results in the amplification of FGR diagnoses within the SGA population, juxtaposed with the insufficient recognition or non-detection of FGR cases in those identified as AGA (53, 54).

Emerging DL approaches have the potential to extract complex features from ultrasound images, enabling more precise diagnosis of SGA compared with conventional methods that depend solely on basic fetal biometry (55). Current AI strategies for fetal growth assessment rely primarily on automated biometric measurements, the incorporation of maternal characteristics, or a combination of ultrasound-derived fetal measurements and maternal factors (4, 56). As an illustration, Mikołaj et al. (57) developed a model derived exclusively from sonographic images, exhibiting a markedly superior sensitivity in the identification of SGA cases compared to Hadlock’s estimations incorporating biometric measurements acquired by experienced physicians The model had been trained with over 433,000 images from over 65,000 examinations, which resulted in a sensitivity of 70% (0.69–0.71) vs. 58% (0.56–0.59) for the Hadlock formula, and a specificity of 91% (57). Despite the DL model outperforming Hadlock formula, its retrospective design and reliance on single-center data raise questions about applicability across diverse populations (57). In another retrospective analysis of second-trimester data, including maternal demographics and ultrasound parameters, ML models predicted SGA at birth with notably higher accuracy (70%) compared to standard clinical guidelines (64%), with UtA-PI and nuchal fold thickness emerging as significant predictors (58). Lastly, in a retrospective cohort of 12,912 pregnancies, a multivariate predictive model integrating third-trimester EFW, AC percentile, maternal characteristics, and first-trimester biomarker levels (PAPP-A and free *β*-hCG), achieved an improved detection rate of 63.5% for SGA at birth (AUC = 0.882), compared to 58.9% when using estimated weight percentile alone (AUC = 0.864) (59).

These findings underscore the potential of AI-driven approaches to enhance early detection of SGA beyond conventional biometry-based calculations. It has been recently shown that DL models applied to large-scale ultrasound datasets can achieve near-expert accuracy in the evaluation of fetal biometry, while maintaining clinically acceptable error margins and offering interpretable outputs, thus supporting more reliable SGA detection (60). Together, these advances illustrate the capacity of AI not only to increase the sensitivity of SGA detection but also to transcend percentile-based classifications by embedding maternal, fetal, and biochemical factors into a precision-medicine framework.

### Fetal macrosomia and large-for-gestational-age (LGA) fetus

Following the common definition, fetal macrosomia occurs if the EFW exceeds 4,000 g, regardless of gestational age (61). It has long been established that early and accurate detection of fetal overgrowth remains challenging because conventional diagnostic methods, primarily ultrasound-based fetal biometry, have limited precision in predicting excessive fetal birth weight, especially in diabetes-complicated pregnancies (62–64). LGA, on the other hand, describes an infant whose EFW is at or above the 90th percentile for its gestational age, hence not necessarily exceeding 4,000 g (65). Although fetal macrosomia and LGA are often used interchangeably, LGA has been shown to perform better as a classifier, whereas macrosomia, defined by crude birth weight, is a better predictor of perinatal morbidity (66–68).

As already mentioned, ultrasound is the primary method used to identify pregnancies at risk of fetal macrosomia or LGA. Evidence from a large cohort study of about 67,000 pregnancies showed that performing an ultrasound between 35 + 0 and 36 + 6 weeks of gestation achieved an AUC of 0.861 (95% CI 0.856–0.867) for detecting LGA fetus (69). Additionally, systematic reviews assessing the accuracy of ultrasound in predicting macrosomia or LGA report positive likelihood ratios of 7.8 (95% CI 5.9–10.5) (70), 5.1 (95% CI 3.0–8.7) (71), and 8.74 (95% CI 6.84–11.17) (72). Collectively, these findings imply that ultrasound has a moderate diagnostic capacity for identifying fetal overgrowth.

Given the aforementioned issues, the application of AI has been increasingly investigated for improving the prediction of excessive fetal weight. AI models predicting macrosomia have demonstrated consistent gains in accuracy when integrating maternal demographics, metabolic parameters, and ultrasound data (50, 73). In a large retrospective study, ML algorithms that integrated maternal characteristics, metabolic parameters, and fetal ultrasound measurements achieved higher predictive performance than standard Hadlock-based approaches, with a specificity of 82% and an AUC of 0.95, demonstrating the value of multimodal data integration in anticipating excessive fetal growth (50). Similarly, a 2025 analysis reported that combining maternal age, body mass index (BMI), parity, and gestational diabetes status together with sonographic parameters yielded superior accuracy for macrosomia prediction compared with biometry alone, thus once again emphasizing the clinical utility of AI in incorporating maternal risk factors into diagnostic models (50). Finally, in a 2023 cohort, an ML model based on maternal clinical characteristics, including age, BMI, and gestational diabetes status, predicted macrosomia with an AUC of 0.88, sensitivity of 83.5%, and specificity of 80.3%, while explainability tools highlighted the strong contributions of pre-pregnancy BMI, maternal weight gain, together with the ultrasound-derived fetal AC measurement (73).

Regarding ultrasound image analysis, a 2024 study demonstrated that convolutional neural networks trained exclusively on fetal scans improved weight estimation in the upper percentiles, thereby increasing the sensitivity of macrosomia detection without compromising specificity (74). Complementary findings from a study by Gu et al. (75) showed that computer vision algorithms could automate fetal measurement processes, outperforming traditional manual sonography in identifying macrosomia and reducing operator variability. Interestingly, in one of the most recent studies, hybrid models combining gated recurrent unit (GRU) networks with attention mechanisms have further improved the prediction of fetal overgrowth directly from ultrasound-derived scans (76).

Similar to FGR/SGA, AI offers the ability to integrate molecular information with maternal and ultrasound data for more precise fetal growth evaluation (24, 77). In this context, Rubini et al. (78) underscored the relevance of paternal biomarkers by showing that homocysteine levels, combined with AI-assisted fetal measurements, correlate with both early and late fetal growth velocity. Comparably, maternal plasma proteins have been investigated by Andresen et al., who demonstrated that multiomics data can be utilized to differentiate AGA from LGA fetuses (79).

**Figure 3:**
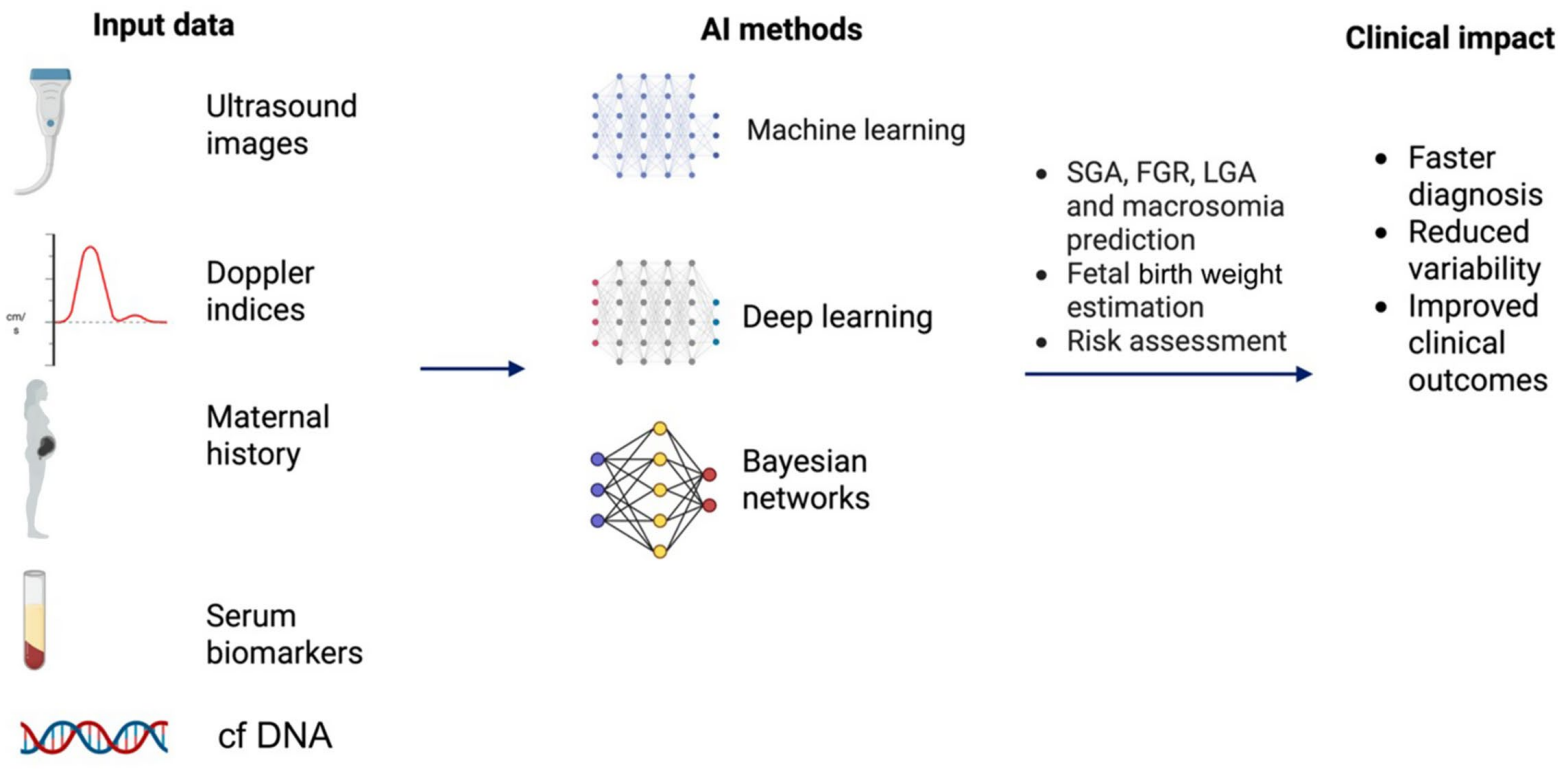
AI integration in prenatal growth assessment. Created with biorender.com. cfDNA, cell-free DNA; FGR, fetal growth restriction; LGA, large-for-gestational-age; SGA, small-for-gestational-age.

To conclude, current investigations illustrate that AI-driven approaches, whether through multimodal risk models, interpretable machine learning, or DL applied directly to ultrasound images, can substantially improve the accuracy, consistency, and clinical trustworthiness of macrosomia/LGA prediction. Integrating clinical, biochemical, and sonographic data captures complex interactions that drive fetal overgrowth, which traditional methods often miss (Figure 3). As a consequence, AI has the potential to outperform standard prediction models, offering the possibility to mitigate complications related to undiagnosed macrosomia or LGA. Nonetheless, despite these advances, prospective validation and cost-effectiveness studies are still lacking, delaying clinical adoption (50, 73).

Given the heterogeneity of study designs and computational methodologies employed across the spectrum of fetal growth disorders, a consolidated overview is essential to differentiate the prevailing strategies. Table 1 summarizes the predominant AI approaches, input data modalities, and validation status for FGR, SGA, LGA, and fetal macrosomia. This comparison highlights the distinct methodological trends, such as the heavy reliance on multimodal Doppler and omics data for detecting pathological growth restriction (FGR), versus the increasing application of deep learning to direct ultrasound imaging for the assessment of fetal size anomalies (SGA and LGA).

**Table 1:** Summary of AI methodologies applied to the diagnosis and prediction of FGR, SGA, LGA, and fetal macrosomia.

Clinical condition	Predominant AI models applied	Input data and selected features	Dataset scale and characteristics	Validation Status
Fetal growth restriction	Machine learning: • Support Vector Machines (41, 51). • Multilayer Perceptron (41) • Decision Trees and Random Forests (48). • Classification via Regression (47). • Logistic Regression (49, 51).	Multimodal integration: • Maternal: age, parity, hypertension, reproductive history (46). • Doppler: uterine artery PI, fetal hemodynamics (47, 48). • Biochemical: sFlt-1/PlGF ratio, placental proteins, metabolites of fatty acid oxidation (42, 48). • Genomic: cfDNA nucleosome profiling and promoter classifiers (49, 51).	Variable: Ranges from small pilot studies (~800 subjects) to large clinical cohorts (>32,000 women) (46, 51).	Mostly retrospective: Most models rely on internal validation (e.g., Leave-One-Out Cross-Validation) (51). Significant lack of external prospective validation (42, 49).
Small-for-gestational-age	Deep learning: •Convolutional Neural Networks (57). • Explainable AI architectures (60). Predictive modeling: • Multivariate regression models (59).	Imaging and biometry: • Direct imaging: Raw ultrasound images/video (pixel-level feature extraction) (57). • Biometry: EFW and AC percentiles (59). • First trimester biomarkers: PAPP-A, free β-hCG (59). • Ultrasound markers: Nuchal fold thickness (58).	Large-scale imaging: Utilizes massive image datasets (e.g., >433,000 ultrasound images) and moderate clinical cohorts (~12,000 pregnancies) (57, 59).	Emerging prospective: Predominantly retrospective, though recent studies have begun prospective, cross-institutional end-user evaluation (60).
LGA and fetal macrosomia	Machine learning: • Standard machine learning algorithms (e.g., Random Forest) (50, 73). Deep learning/hybrid: • Convolutional neural networks (trained on fetal scans) (74). • Gated recurrent unit with attention mechanisms (76). • Computer vision algorithms (75).	Metabolic and anthropometric: • Maternal metabolic: Pre-pregnancy BMI, gestational diabetes status, maternal weight gain (73). • Fetal: AC measurements, soft tissue analysis (73, 75). • Omics: Maternal plasma proteins, paternal homocysteine levels (78, 79).	Retrospective clinical data: Studies utilize large retrospective cohorts to capture sufficient macrosomia cases (73). Large ultrasound datasets used for specific weight estimation tools (75).	Proof-of-concept: High predictive performance (AUC ~ 0.88–0.95) reported in retrospective testing (50, 73), but lacks broad prospective validation and cost-effectiveness analysis (73).

AC, abdominal circumference; BMI, body mass index; cfDNA, cell-free DNA; EFW, estimated fetal weight; FGR, fetal growth restriction; free β-hCG, free beta chorionic gonadotropin; LGA, large-for-gestational-age; PAPP-A, pregnancy-associated plasma protein A; PI, pulsatility index; PlGF, placental growth factor; sFlt-1, soluble fms-like tyrosine kinase-1; SGA, small-for-gestational-age.

### Challenges and ethical considerations

Despite promising results, several limitations must be acknowledged before AI-based models analyzing fetal growth abnormalities can be widely implemented in clinical practice. Firstly, numerous published studies are retrospective, single-center, and underpowered, which limits generalizability and reproducibility. Overfitting, where algorithms perform well in the training cohort but fail to extrapolate to broader populations, remains a significant barrier to clinical translation. Ensuring external validation across diverse ethnic, socioeconomic, and healthcare settings is crucial, yet difficult due to the limited availability of representative datasets.

Bias is another major concern. In the context of fetal growth, models trained predominantly on data from high-income countries may not perform adequately in low-resource settings, potentially worsening health inequities rather than reducing them. The lack of standardized reporting and transparent model development further complicates the evaluation of fairness and accountability.

Technical and infrastructural limitations pose additional challenges. High computational requirements, insufficient integration with existing ultrasound equipment, and limited technical expertise in many healthcare facilities may hinder implementation. Even in tertiary centers, investments in hardware, software, and personnel training will be substantial. Without appropriate funding and structured training programs, disparities between well-resourced and under-resourced centers may widen, counteracting the intended benefits of AI.

From an ethical standpoint, the protection of patient data is paramount. AI-driven approaches often require the collection and storage of sensitive maternal and fetal information, including imaging, genetic, and clinical data. Ensuring robust data security and compliance with local and international privacy regulations is essential to maintain patient trust. Additionally, unresolved questions regarding liability, whether responsibility lies with clinicians, hospitals, or developers in the event of diagnostic errors, necessitate clear legal frameworks before routine clinical deployment.

Finally, the issues of cost-effectiveness and sustainability must be addressed. While AI has the potential to reduce costs by streamlining workflows and enhancing diagnostic accuracy, large-scale prospective trials are required to determine its real-world impact and economic feasibility. Without such evidence, integration into clinical guidelines remains questionable.

### From bench to bedside: pathway for clinical translation

In spite of favorable findings, most AI models designed for the diagnosis and management of fetal growth disorders remain at a proof-of-concept or retrospective validation stage. For successful translation into clinical practice, several steps are required: (1) Prospective, multicenter validation across diverse populations to confirm reproducibility and generalizability; (2) Integration with common ultrasound platforms to enable real-time, operator-independent application; (3) Regulatory approval by agencies such as the FDA and EMA, requiring standardized reporting of performance and safety; (4) Implementation studies assessing cost-effectiveness, workflow impact, and acceptability by clinicians and patients. Only through this pipeline can AI shift from experimental use to becoming a routine component of precision perinatal medicine.

## Conclusion

AI shows strong potential to transform the detection and management of fetal growth disorders, achieving clinician-level accuracy alongside greater efficiency. By integrating biometric, maternal, and imaging data, AI may enable earlier diagnosis, personalized monitoring, and broader access to quality prenatal care. Nevertheless, AI translation into daily practice will depend on large-scale validation, robust infrastructure, and clear ethical and legal frameworks. If these challenges are addressed, AI could become the cornerstone of precision perinatal medicine, significantly improving maternal–fetal outcomes.
