# Correction: Application of artificial intelligence in diagnosis and management of fetal growth disorders: a comprehensive review

**DOI:** 10.3389/fmed.2026.1792399

**Published:** 2026-02-02

**Authors:** Frontiers Production Office

**Affiliations:** Frontiers Media SA, Lausanne, Switzerland

**Keywords:** artificial intelligence, fetal growth disorders, fetal growth restriction, fetal macrosomia, intrauterine fetal growth restriction, large-for-gestational-age, small-for-gestational-age

Figure 3 was erroneously omitted at publication. The corrected Figure 3 with caption appears below.

**Figure 3:**
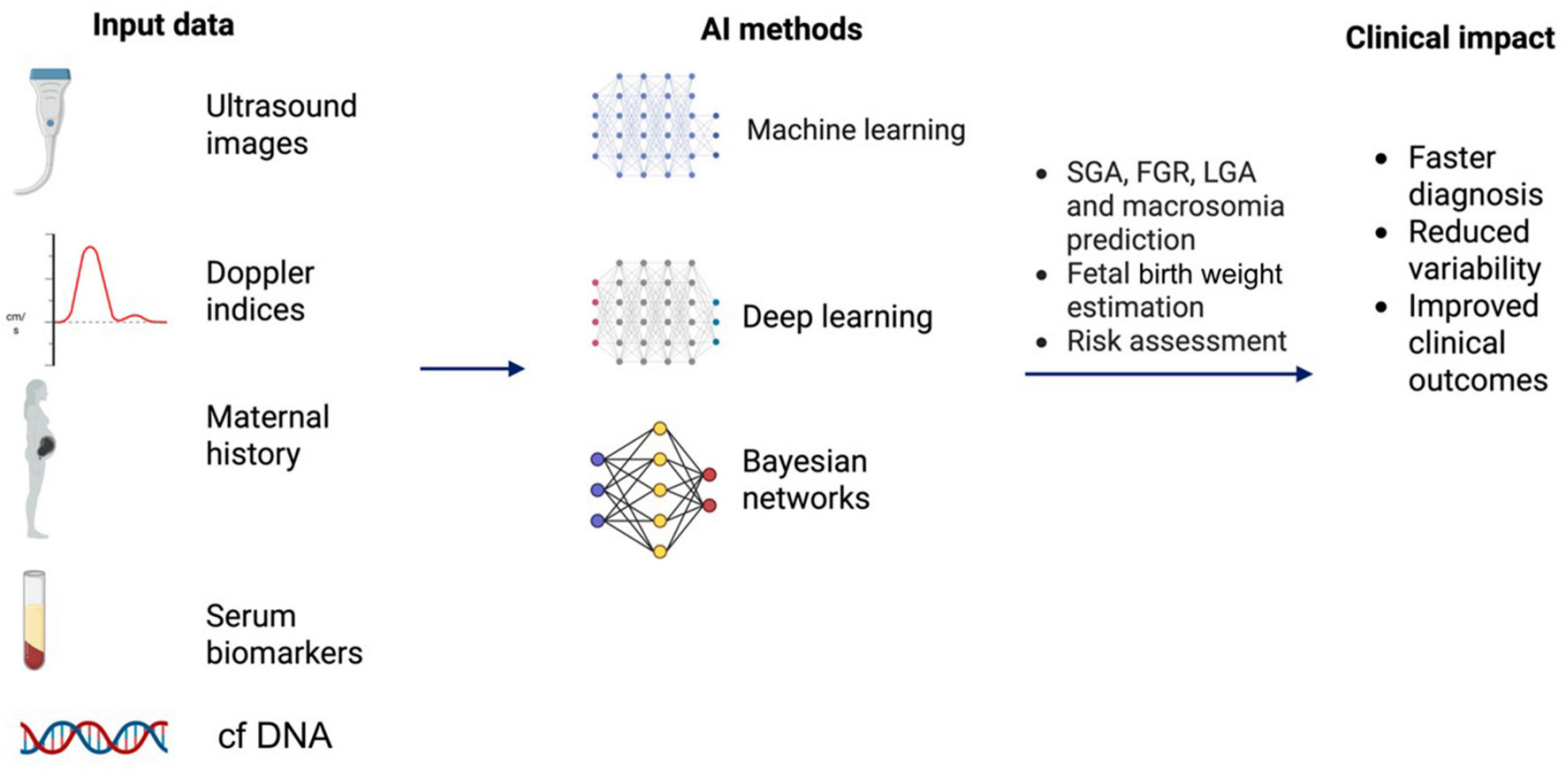
AI integration in prenatal growth assessment. Created with biorender.com. cfDNA, cell-free DNA; FGR, fetal growth restriction; LGA, large-for-gestational-age; SGA, small-for-gestational-age.

Figure 3 was not cited in the article. The citation has now been inserted in the section **Materials and methods**, subsection *Fetal macrosomia and large-for-gestational-age (LGA) fetus*, Paragraph Number 6 and should read:

“Integrating clinical, biochemical, and sonographic data captures complex interactions that drive fetal overgrowth, which traditional methods often miss (Figure 3).”

The original version of this article has been updated.

